# Understanding the Design Elements Affecting User Acceptance of Intelligent Agents: Past, Present and Future

**DOI:** 10.1007/s10796-021-10230-9

**Published:** 2022-01-04

**Authors:** Edona Elshan, Naim Zierau, Christian Engel, Andreas Janson, Jan Marco Leimeister

**Affiliations:** 1 Institute of Information Management, University of St. Gallen, St.Gallen, Switzerland; 2 Information Systems, Research Center for IS Design (ITeG), University of Kassel, Kassel, Germany

**Keywords:** Human, Computer interaction, Intelligent agent, Information systems, Research agenda, Systematic literature review

## Abstract

Intelligent agents (IAs) are permeating both business and society. However, interacting with IAs poses challenges moving beyond technological limitations towards the human-computer interface. Thus, the knowledgebase related to interaction with IAs has grown exponentially but remains segregated and impedes the advancement of the field. Therefore, we conduct a systematic literature review to integrate empirical knowledge on user interaction with IAs. This is the first paper to examine 107 Information Systems and Human-Computer Interaction papers and identified 389 relationships between design elements and user acceptance of IAs. Along the independent and dependent variables of these relationships, we span a research space model encompassing empirical research on designing for IA user acceptance. Further we contribute to theory, by presenting a research agenda along the dimensions of the research space, which shall be useful to both researchers and practitioners. This complements the past and present knowledge on designing for IA user acceptance with potential pathways into the future of IAs.

## Introduction

Against the backdrop of the steep technological advancements in algorithms, data storage, and computing power during the last decades (von Krogh, 2018), which have facilitated the rise of Artificial Intelligence (AI), intelligent agents (IAs) are permeating both business and society. Thus, it is not surprising that IAs have sparked the interest of both researchers and practitioners in recent years (Pfeuffer et al., 2019). IAs can be described as agents that perceive and respond in a timely manner, are capable of interacting with other agents (i.e., humans), and react to their environment (Rudowsky, 2004). With their capabilities, these agents are revolutionizing how machines are interacting with users in natural language (Janssen et al., 2020) and thus allow applications in a wide array of fields. For instance, IAs are facilitating assistance in hands-free contexts such as clinical surgery or for complex manual assembly tasks (Laumer et al., 2019) and have changed the way people order products (Kushwaha & Kar, 2021), ask for the way, and check the weather (Kendall et al., 2020). Thus, they herald a huge potential for digital disruption in both organizational processes and user-based processes through the humanization of human-computer interaction (Porra et al., 2020). Therefore, these agents represent a novel type of Information Systems (IS) entity that can be distinguished from other IS entities by their high level of interaction and intelligence (Maedche et al., 2019). These capabilities have a significant impact on user perceptions and raise novel theoretical and design-related issues, the most prominent of which revolves around an emergent conversation-based interaction paradigm (e.g., Clark et al., 2019). However, the transition to IAs exacerbates several challenges in the area of users’ acceptance, which necessitates additional research (Pfeuffer et al., 2019) and has sparked a vivid scientific discourse.

As a result, in recent years, a diverse body of empirical work on IAs has emerged in a variety of disciplines, most notably in the Information Systems (IS) and Human–Computer Interaction (HCI) domains (Janssen et al., 2020). Here, researchers have investigated IAs based on a variety of user interaction outcomes (e.g., attitudes, perceptions, intentions, and behavior as for instance, in Lee et al., 2020). Furthermore, research in this vein has examined the effects of a plethora of design elements provided by IA interfaces on these interaction outcomes (Feine et al., 2019; Janssen et al., 2020). Consequently, these substantial research efforts have led to an ever-growing number of relationships between independent and dependent variables, building on existing theories that aim to explain and predict the nature of IAs. However, the variables investigated in these studies are heterogeneous. Thus, summarizing, analyzing, and evaluating findings from the overall body of empirical research on IAs is difficult. As demonstrated in this paper, this results in a fragmented and sparse literature base, as well as sometimes even contradictory research results.

Consequently, several literature reviews and meta-studies (e.g., taxonomic classifications of IAs; Janssen et al., 2020; Zierau et al., 2020) have emerged in recent years. For example, these focus on specific sub-classes of IAs, such as pedagogical agents (MØrch et al., 2005), or on literature on AI-based applications in general (Rzepka & Berger, 2018), whereas classification-based papers concentrate on the structural characteristics of IAs (Janssen et al., 2020; Nißen et al., 2022). However, a merely domain-specific scope, as well as a too high level of abstraction of such reviews, lead to research is still being dispersed across various research streams, thus lacking an axis of integration. As a result, the scientific and practical knowledge that has grown dramatically in recent years, as demonstrated by this review, remains separated. Therefore, work is needed to leverage on the synergies of integrating research insights that highly interdisciplinary fields such as HCI and IS need for advancing their body of knowledge. Especially in relatively new research fields, such as IA research, an integrated conceptualization and synthesis of representative literature are required, upon which future research efforts can be built (Torraco, 2005).

So far, to the best of our knowledge, such an integrated conceptualization does not exist, resulting in terminological ambiguity and a lack of construct clarity (Suddaby, 2010) in IA research. As a result, we intend to encapsulate the rapidly expanding empirical body of knowledge on IAs into a concise and meaningful manner that is useful to researchers. Therefore, the following research question (RQ) is addressed in this paper:

- RQ: *What is the current state-of-the art of empirical research on user interactions with intelligent agents?*

To answer this question, we examined 107 empirical publications within the scope of the current study that fell under the umbrella of IS and HCI research. Thus, we examined the literature published in 20 seminal outlets. We analyzed a plethora of findings from a variety of studies and compiled the findings from both quantitative and qualitative empirical research. Furthermore, we extracted the most frequently studied constructs from the review and used them to develop three descriptive models. These models both represent the current state-of-the-art in IA research and help to identify white spots in empirical research on IAs. These indicate potentially fruitful avenues for future research, which we structure in an integrated research agenda. Researchers will be provided with conceptually sound and empirically grounded starting points for expanding the body of knowledge on IAs in HCI and IS research. The paper concludes with a conclusion and a discussion of the study’s limitations.

## Conceptual Foundations of Intelligent Agents

The scientific and industrial interest in IAs has grown significantly in recent years (e.g., Feine et al. 2019; Pfeuffer et al. 2019). The groundwork for the new technology was laid in 1966 when Joseph Weizenbaum created a computer program that communicated with humans via a text-based interface and passed the touring test (Weizenbaum, 1966). In the 1980s, these text-based interfaces were followed by the development of voice-based dialogue systems and embodied conversational agents (McTear et al., 2016). A number of overlapping trends have contributed to the increased interest in this system type. On the one hand, new generations of IAs have emerged as a result of recent advancements in AI, particularly in natural language processing, that can be used to augment an increasing number of tasks such as hands-free surgery assistance in healthcare (Laumer et al., 2019), assisting in homework in education (Winkler et al., 2019), and making customer service available 24/7, 365 days a year through chat and voice bots (Qiu & Benbasat 2009). Conversely, the conversational nature of IAs enables new and potentially more convenient and personal ways of accessing content and services, ultimately improving user interactions with IS (Følstad & Brandtzaeg, 2020). Along with these advancements, there has been an increase in the scientific interest in how these interfaces affect user perceptions. Numerous studies have been conducted in recent years under the terms of *Intelligent Personal Assistant* (Hauswald et al., 2016)*, Smart Personal Assistant* (Knote et al., 2020), *Chatbot* (Følstad & Brandtzæg, 2017), and *Conversational Agent* (Feine et al., 2019). As the overarching topic of this paper, we will highlight some key features of IAs.

According to Maedche et al. (2019), IAs are distinguishable from other entities of IS due to their capabilities for *interaction* and *intelligence*. Regarding the first dimension, the ability to engage with users via natural language is formative to our understanding of IAs (Feine et al., 2020). Typically, IAs have relied on rigid behavioral patterns. Those agents could only respond to simple requests by matching user inputs against a set of stored patterns (McTear et al., 2016). Novel forms of IAs, on the other hand, can now process compound natural language and thereby respond to increasingly complex user requests (Knote et al., 2018). One example is Amazon’s Alexa, which assists users in carrying out daily tasks through an advanced voice interface, eventually serving as their personal assistant (Benlian et al., 2019). These agents are increasingly mimicking human-to-human interaction (Feine et al., 2019; Purington et al., 2017), allowing for a more convenient and natural way to interact with technology (Knote et al., 2019). Furthermore, modern IAs are now typically distinguished by an intelligence component (Maedche et al., 2019). For the purpose of this paper, we refer to intelligence as “*the ability to accomplish complex goals, learn, reason, and adaptively perform effective actions within an environment*” (Dellermann et al., 2019, p. 638), which broadly speaking is the capacity of an entity for the acquisition and application of knowledge (Gottfredson, 1997). This property makes IAs more adaptable to different users and given context situations. Thus, IAs are capable of “*learning”* how to use inputs such as environmental data and user preferences (Maedche et al., 2019). IAs can adapt and personalize their behavior over time by drawing on a constantly growing data set, resulting in autonomous characteristics (Pfeuffer et al., 2019). In this paper, we consider a wide range of agents, including less advanced agents (i.e., rule-based or scripted agents), in order to provide a comprehensive overview of respective user interactions.

Essentially, these capabilities may have a significant impact on user interactions with these systems, raising several questions about the theoretical foundations and design elements of IAs. In this regard, it has been demonstrated that the human-like characteristics of IAs may cause users to exhibit emotional, cognitive, or behavioral reactions resembling human interactions (Krämer et al., 2005). Hence, researchers are increasingly relying on the *Computer Are Social Actors* (CASA) paradigm as their theoretical foundation to explain specific user behaviors.

Accordingly, humans identify with certain IA design elements (e.g., an avatar), which causes them to categorize a technical system as a relevant social entity (Nass et al., 1994). In this context, design elements are the distinguishing technical, contextual, and knowledgeable features that frame the IA (Janssen et al., 2020). Recently, the inventory of potential design elements for both verbal and nonverbal communication has grown significantly (Feine et al., 2019), allowing IA designers to address common user concerns (e.g., lack of trust) and create increasingly convincing user interaction experiences (Pfeuffer et al., 2019). Simultaneously, a slew of research has emerged in various disciplines, most notably in the IS and HCI domains, that empirically investigates the impact of specific IA design elements on various user perceptions. As a result, most studies have concentrated on one or a few design elements or configurations and their impact on selected user perceptions, resulting in a fragmented literature base and sometimes contradictory research findings. An integrated analysis aggregating empirical insights on the diversity of IA design elements could address this shortcoming, increasing our understanding of user behaviors and assisting us in identifying future research needs.

## Prior Literature Reviews on Intelligent Agents

In this section, we summarize prior IAs literature reviews. In particular, we were able to identify five major reviews of IA literature, which we discuss here in order to determine how the review at hand differs along several dimensions (see Table 1). This will aid in clearly defining the contribution of this paper.

**Table.1:** Overview of prior reviews

Author(s)	Time period of review	Sample size	Scope	Review-focus
Bavaresco et al. (2020)	2009–2019	58 articles	IS and HCI conferences and journals	Literature published in the last decade that focuses on market viewpoints such as sectors, goals, and challenges of conversational agents in the business domain
Rapp et al. (2021)	2010–2020	83 articles	Scattered across HCI, medicine, psychology	Map the recurring themes, describing how people experience and what kinds of drawbacks can be observed in human-chatbot conversations
Rheu et al. (2021)	Up to 2019	29 articles	HCI	Focus solely on trust-enhancing design elements
Rzepka and Berger (2018)	Up to 2018	96 articles	14 IS & HCI-Outlets	Adoption characteristics of AI-based conversational agents (System, User, Task & Context, Interaction)
Van Pinxteren et al. (2020)	1999–2018	61 articles	Various field, not only IS or HCI	The effects of conversational agents’ communicative behaviors on relational outcomes were investigated in service encounters
This review	1996–2020	107 articles	20 IS & HCI-Outlets	We code the independent and dependent variables, as well as their relationships, to summarize the empirical academic literature. The paper also discusses knowledge gaps

Existing reviews on IAs either assume an overall perspective on AI-based technologies (e.g., Rzepka & Berger, 2018), which appears to be arguably too broad to draw meaningful conclusions from user interaction based on the specific characteristics of IAs (i.e., too high level of abstraction), or they focus on specific application domains such as education (e.g., Winkler & Söllner, 2018) or business (Bavaresco et al., 2020), which appears to be too narrow in scope to draw overall conclusions on user interaction (Pfeuffer et al., 2019). For example, Van Pinxteren et al. (2020) focused on human-like communicative behaviors that had previously been studied in conversational agents, as well as their effects when it comes to service encounters. Furthermore, to the best of our knowledge, there is no review that takes a distinct perspective on the empirical effects of IAs, despite the accelerating and, at the same time, fragmented growth of practical and scientific knowledge in this area (Janssen et al., 2020). As a result, we address the lack of an integrative perspective by conducting a systematic literature review of the empirical literature on IAs in order to identify validated findings and research gaps.

## Research Approach

Hereafter, we describe our research approach to review empirical IA literature, which was informed by the methodological approach employed by Jeyaraj et al. (2006). To that end, we followed the steps for identifying, coding, validating, and analyzing quantitative and qualitative empirical findings.

### Paper Selection Process

To identify relevant literature as the basis for the state-of-the-art analysis, we conducted a systematic literature review (SLR) following Webster and Watson (2002) and vom Brocke et al. (2015). The overall scope of the conducted SLR can be defined along the dimensions of process, source, coverage, and techniques of the SLR (vom Brocke et al., 2015). Based on a *sequential search process,* we searched relevant journals and conference proceedings from the field of IS and HCI literature as a *source*. Thereby, our literature search intends to reach a *representative coverage* of the design elements reported in the literature. Thus, to establish the basis for our analysis, we used a *comprehensive set of techniques* (i.e., keyword search, backward and forward search). To reach a high level of reproducibility and transparency of our research, we will describe the three single methodical steps that we undertook.

In the *first step*, we selected the search strings. Since we aimed to identify a wide range of literature on IAs, the search string was chosen to be rather broad. Based on recent publications, we identified different keywords that researchers used to describe IAs. This resulted in the following search string:

*“conversational agent” OR “intelligent agent” OR “chat bot” OR “chatbot” OR “dialogue system” OR “smart personal assistant” OR “smart assistant” OR “intelligent assistant”.*

In the SLR, we used all variations of the keywords – singular, plural, hyphenated, or not hyphenated. In the *second step*, we selected the outlets. As our goal was to identify representative literature samples of different empirical research perspectives on user interaction with IAs, our search covered multiple journals and conference proceedings. We chose this approach because journal acceptance processes take substantially longer than conference proceedings to be processed, which would have led to neglecting some of the most relevant literature since IA research represents a young and nascent topic. For the selection of outlets, we identified two broad areas for deriving design elements of IAs – IS and HCI – as they cover a substantial share of literature on IAs.

Suitable journals and conference proceedings at the intersection of HCI and IS that provided an overview of high-quality and relevant research in the respective research fields were selected using both the AIS Senior Scholars’ Basket, and relevant IS journals and conferences based on the recommendations of the special interest group on human–computer interaction. Moreover, to safeguard the relevance of our results, we discussed our selection of journals and conference proceedings with two senior researchers from the field of interest who were not involved in the writing process of the paper. Based on these inputs and their feedback, we selected 20 journals and proceedings for our keyword search, as seen in Table 2. Finally, in the *third step,* we selected the papers. We searched in the title, abstract, and keywords of the papers as we assumed that papers that deal with design element of IAs as a focal unit of analysis would exhibit the search strings defined above there. The outlet-based search revealed 383 hits. This number contained literature not relevant to this paper. In an initial screening process, the identified papers were analyzed based on their abstracts. We only included papers that referred to any type of IAs and that provided empirical insights on user interaction with IAs. Papers dealing with this topic trivially or marginally, such as those generally dealing with technology acceptance of IAs, were removed from the sample. This resulted in a selection of 76 publications. Finally, we also performed a forward and backward search to capture papers not covered through the database search. Through screening the references and applying forward searches using Google Scholar, 31 articles were added to the list, resulting in the final number of 107 relevant papers.

**Table.2:** Overview of searched journals and conference proceedings

Field	Outlets	Total hits	Relevant hits
**Information Systems**	ACM Transactions on Information Systems	16	0
	Decision Sciences	6	0
	Decision Support Systems	39	5
	European Journal of Information Systems	6	0
	Information Systems Journal	2	0
	Information Systems Research	6	0
	Journal of Information Technology	1	0
	Journal of Management Information Systems	21	1
	Journal of the Association for Information Systems	1	0
	Proceedings of the International Conference on Information Systems (ICIS)	14	2
	Proceedings of European Conference on Information Systems (ECIS)	3	0
**Human-Computer Interaction**	ACM Transactions on Computer-Human Interaction	17	6
	Human–Computer Interaction	12	3
	International Journal on Human–Computer Studies	43	6
	Journal of Computer-Mediated Communication	6	0
	Journal of the ACM	1	0
	User-Modelling and User-Adapted Interaction	17	2
	Proceedings of the Conference on Human Factors in Computing Systems	172	51
Overall Hits		383	
Relevant Hits		76	
Additional Papers through Backward & Forward Search		31	
**Relevant Papers for Analysis**		**107**	

### A Frame for Paper Analysis

We aim to use a theoretically sound frame for guiding the analysis of the retrieved papers. The types and effects of factors affecting user acceptance of IAs differ depending on the theoretical lenses used by the researchers. Hence, we briefly explain our reasoning for selecting our frame of analysis.

First, we present the scaffolding model developed by Wirtz et al. (2018) for the purpose of grounding the analysis of dependent variables on a solid theoretical basis. Then, we elaborate our procedure for the independent variables, for which we seize a taxonomic classification of the social cues of CAs introduced by Feine et al. (2019). This shall allow establishing exhaustive and exclusive paper analysis results benefitting the clarity and structure of the field.

In the vein of dependent variables, most studies share similar theoretical foundations, which are primarily based on the Technology Acceptance Model (TAM) (Davis, 1989) and its subsequent modifications, such as the TAM2 (Venkatesh & Davis, 2000), TAM3 (Venkatesh & Bala, 2008), Unified Theory of Technology Use and Acceptance (UTAUT) (Venkatesh et al., 2003), and UTAUT2 (Venkatesh et al., 2012).

**Fig. 1:**
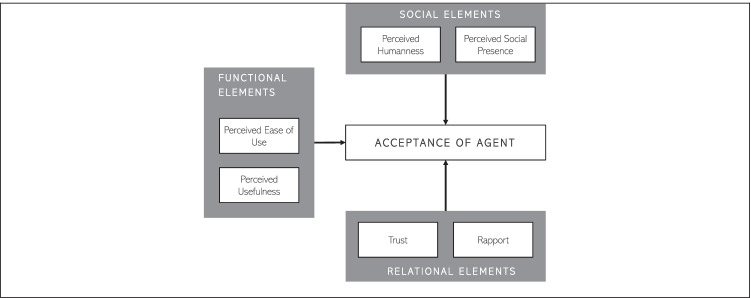
Agent acceptance model adopted by Wirtz et al. (2018)

**Fig. 2:**
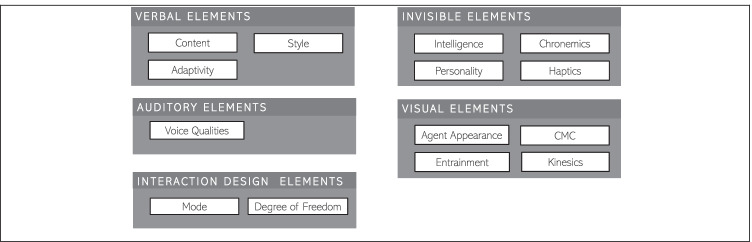
Taxonomy of social cues adopted by Feine et al. (2019)

However, although literature often refers to such renowned theories due to their adaptability and accessibility, their utility is largely context-specific (Lowe et al., 2019), and they may not be comprehensive enough to demonstrate the introduction of emerging technologies such as IAs (McLean & Osei-Frimpong, 2019). As a result, introducing them in a new context necessitates a close examination of its fundamental elements as well as empirical confirmation of key relationships. Furthermore, IAs are more personalized, linked, and open than previous technologies (Gummerus et al., 2019). Thereby, user acceptance of IAs shall be determined not only by their practical efficiency but also by their capacity and skills to meet social-emotional and relational needs (Lee et al., 2020; van Doorn, et al., 2017; Wirtz et al., 2018). Wirtz et al. (2018) developed the Service Robot Acceptance Model (sRAM), which expands on the original TAM by including social-emotional and relational variables. The model depicted in Figs. 1 and 2 views service robots as “*[…] system-based autonomous and adaptable interfaces that interact, communicate and deliver service to an organization’s customers*” (Wirtz et al., 2018, p. 909). This notion can be transferred and adapted to the context of IA acceptance as IAs are described as entities that perceive and respond in a timely manner, are capable of interacting with other agents (i.e., humans), and react to their environment (Rudowsky, 2004).

Per the underlying assumption of sRAM, user acceptance of IAs is determined by how well IAs meet functional, socio-emotional, and relational requirements (Davis, 1989; Fiske et al., 2007; Solomon et al., 1985).

The sub-dimensions of social elements included in the sRAM model are *perceived humanness*, *perceived social presence,* and *social interactivity*. In this regard, *social presence* can be defined as “*the extent to which other beings in the world appear to exist and react to the user*” (Heeter, 1992). Whilst the *perceived humanness* refers to the distinguishability of an IAs from a human (Wuenderlich & Paluch, 2017). Here it must be noted that we did not include the sub-dimension *social interactivity* within our analysis.

Aside from social aspects, two important *relational elements* have been identified, i.e., *trust* and *rapport*. *Trust* is usually defined as an expectation that another entity “will perform, a particular action important to the trustor [i.e., user], irrespective of the ability to monitor or control that other party [i.e., IA]” (Mayer et al., 1995, p. 712). The sub-dimension *rapport* can be defined as the user’s perception of a pleasant encounter with an IA (i.e., IA being friendly, the IA’s ability to stimulate interest, and meeting the user’s needs for fulfillment of a task) as well as a personal relationship between the user and the IA. In general, it can also be described as the personal interplay between two parties (Gremler & Gwinner, 2000).

The functional elements included in sRAM are the original dimensions of the TAM model (Davis, 1989): *subjective norms, perceived ease of use,* and *perceived usefulness.* A user’s views about what other (important) users think they should do (or not do) in a particular scenario are referred to as *subjective norms* (Fishbein & Ajzen, 1977; Venkatesh & Davis, 2000). These social conventions may have a positive relationship with the acceptance of new technologies because people are more likely to respond in a certain way if they assume it is acceptable by society. In previous research, the *subjective norm* played an inconclusive role and led to divergent views (Schepers & Wetzels, 2007). In addition, IAs are a rather new technology, therefore, we do not know how users’ might adopt it to enhance their social status quo (i.e., McLean & Osei-Frimpong, 2019). Within the sub-dimension, *perceived usefulness,* we refer to the extent to which a user believes that using a specific system will improve its performance of fulfilling a task (Davis, 1989). Whereas the *perceived ease of use* refers to “*the degree to which a person believes that using a particular system would be free of effort*” (Davis, 1989).

As a result, we use this slightly modified version of the sRAM model as a guideline in our coding and analysis of IA design dependent variables.

To facilitate the discussion of the high quantity of independent variables, we categorized them into aggregated dimensions based on a taxonomic classification of the social cues of CAs introduced by Feine et al. (2019), which we extended based on our coding of selected categories, as some design elements did not fit these categories (i.e., *interaction*). Furthermore, we did exclude some of the original categories, as they were not fitting for our analysis frame (i.e., *vocalizations*).

Having a look at the *verbal elements*, we refer to this category for all IA elements that can be expressed by words, either written or spoken (Antaki, 2008). This category includes the sub-categories *content, style,* and *adaptivity*. In accordance with Feine et al. (2019) and Collier & Collier (2014), *content* focuses on what is being said and *style* on how something is being said. In comparison, *adaptivity* concentrates on the IA’s verbal adoption to the user’s *style* (e.g., Akahori et al., 2019). This dimension was added to the original taxonomy by Feine et al. (2019).

Elements that are included within the *auditory* category refer to design elements that can be perceived via the sense of hearing except the words themselves (Burgoon et al., 2013). The original taxonomy of social cues included two sub-categories, *voice qualities,* and *vocalizations*. Whilst the category *voice qualities* refers to all the elements that represent permanent and adjustable characteristics of the voice, such as pitch, volume, or the rate of the speech (Schmitt et al., 2021). We did not include the subcategory vocalizations in this paper’s analysis because there were not enough findings in this subcategory. However, because voice is becoming an increasingly important channel for IAs (i.e., Kendall et al., 2020), future research should focus on the empirical evidence pertaining to this sub-category.

The design elements in the category *interaction* allude to the interaction’s underlying structural representation, both in terms of communication medium and turn-taking mechanism. Within this area, we refer to design elements pertaining to *mode* and *degree of freedom*. It should be noted that the category of interaction design components is not included in the original taxonomy of social cues (Feine et al., 2019). We added this category since we found a lot of empirical research that focused on the mode of the IA, and the study by Feine et al. (2019) did not include a review of interaction design aspects. In this sense, the sub-category *mode* refers to the mode of interaction, such as chat or voice. Whereas the *degree of freedom* includes how free the user is in their engagement with the IA. We hope to gain a better grasp of associated interaction design consequences by expanding the original taxonomy of social cues.

Within the original taxonomy of social cues, the category *invisible elements* included the sub-categories *chronemics* and *haptics*. In the realm of our study, we adapted the original taxonomy and added the sub-category *intelligence* as well as *personality*, since both can be described as design elements that cannot be perceived by the sense of hearing or seeing (Knapp et al., 2013). The elements within the four sub-categories are also referred to as the “*silent language*” (Hall, 1990). In this context, the sub-category *chronemics* is referring to as timing-related cues in communication and thus are also related to turn-taking. While haptics can be described as tactile communication (Leathers & Eaves, 2015), and include the perception of touches such as high fives, kisses, or slaps. Even though they are visible in the sense of the eye of being able to sense them, they “*communicate powerful meanings in the absence of any illumination and […] the decoder relies on cutaneous receptors rather than eyesight to decode them*” (Leathers & Eaves, 2015, p. 13). Similar to the sub-category *haptics*, also the sub-category *personality* might be “visible” from time to time, however, within this study, it is being classified as an invisible design element. In this sense, the sub-category *personality* refers to enduring dispositions that are relatively stable over time, e.g., hard-working, calm, emotional (Goldberg, 1990). When it comes to *intelligence*, we follow as previously described the definition of Dellermann et al. (2019) and define it as the ability to achieve difficult goals, learn, reason, and perform effective behaviors.

Lastly, the category of *visual* design elements encompasses all non-verbal design elements that are not invisible and can visually be perceived except the words themselves (Leathers & Eaves, 2015). This category can be distinguished into four sub-categories *agent appearance, computer-mediated communication (CMC), kinesics,* and *entrainment*. The latter sub-category was named *proxemics* in the original taxonomy of social cues and was adapted for the means of this study. In accordance with Cauell et al. (2000), we describe *entrainment* as the adjustments of visual elements to the user, ensuring that the conversation will proceed efficiently. Whilst the *agent appearance* can be described as the IA’s graphical representation (Burgoon et al., 2013; Feine et al., 2019), *kinesics* refer to all body movements of an IA in the case of an embodied representation (Burgoon et al., 2013; Feine et al., 2019). The sub-category *CMC* refers to visual elements that augment or modify written texts, such as emojis or typos (Kalman & Gergle, 2014; Rezabek & Cochenour, 1998; Walther, 2006).

Using the sRAM model and the taxonomy of social cues as a frame for analysis serves as a guiding lens for the subsequent coding of the papers in the paper analysis step.

### Paper Analysis

The 107 relevant papers were analyzed from a concept-centric perspective using an abductive approach. Therefore, we followed an iterative process aggregating the insights from identified studies, which required multiple coding rounds of the identified papers by different researchers. Thereby, the iterative process was started by two of the researchers to independently code a subset of 20 randomly chosen articles. For each of the 20 studies, we listed each dependent and independent variable as named by the author(s), which together formed our initial list of *author variables* (e.g., *delayed responses* and *more human-like* in Gnewuch et al. (2018, p. 11)).

Using the sRAM model and the taxonomy of social cues as a guiding frame for analysis, we carried out selective coding to create a comprehensive allocation of codes to our set of articles (Corbin & Strauss, 2014). In that, the sRAM model and the taxonomy of social cues informed the development of superordinated categories that we used for paper analysis. Moreover, we captured contextual variables such as the application domain and task of the IA.

Next, we re-examined the initial subset set of 20 articles and mapped author variables to our superordinated categories. During the next iteration, two researchers independently coded another subset of 20 articles. Thereby, we coded the dependent, independent, and structural variables and mapped these variables to the superordinated categories (e.g., *delayed responses* to *chronemics* and *more-human like* to *perceived humanness* in Gnewuch et al. (2018)). Afterward, these researchers discussed their own independent findings. In case the respective findings differed, a third researcher was involved in discussing the differences. This process was concluded once all articles were coded.

Concurrent with the aggregation of the codes from open coding, we also coded for empirical relationships between an independent and a dependent variable in each study. Thereby, following Jeyaraj et al. (2006), we assigned four possible codes to the relationship between independent and dependent variables: *″+1″*, *″−1″*, *″0″* and “*M*”. In this process, we coded *″+1″* for a positive relationship, *″−1″* for a negative relationship, and *“0″* for relationships that were studied but did not show any significant value in the empirical results. In quantitative studies, we used P < 0.10 as the requirement for a significant positive or negative relationship. In case the study was qualitative, we relied on the authors’ argumentation, signified by a robust theoretical anchoring, which we coded as *“M”*. All told, we coded 389 relationships between independent and dependent variables (e.g., +1 for the relationship between *turn taking* and *conversation flow* in Winkler et al. (2019)).

## Results

**Fig. 3:**
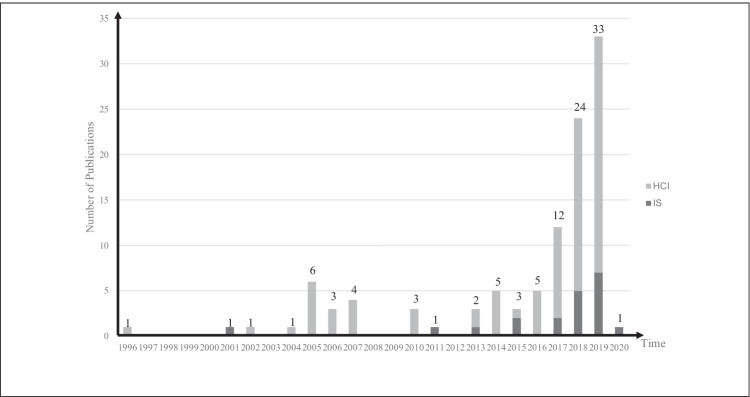
Number of publications over time (n = 107, included in analysis of findings)

Figure 3 shows that the number of identified publications has been steeply growing during the last years. The youngest paper was from 2020, and the oldest paper was from 1996. The majority of the papers were published within the last four years, which supports our initial assumption that IAs represent an emerging research field. This is also reflected by the fact that most of the papers were from conference proceedings, which gives testament to the relative youth of the field. Moreover, it is worth noting that a majority of the investigated papers were from the HCI discipline (87 papers), while publications in IS outlets (20 papers) are only recently directing attention towards IAs. The first contributions were rather explorative, incorporating a multitude of investigated variables, while recent papers were more specific concerning the theoretical lenses applied and the effects investigated. Further, the examined contributions included empirical data from various application contexts and data sources. Of the 389 coded relationships between independent and dependent variables, 213 were positive and significant, 52 were negative and significant, 87 relationships showed no significant relationship. The overrepresentation of positive effects on our dependent variables can either be explained by a strong focus on positive effects or by the resistance of researchers to report negative effects regarding user interaction with IAs. Furthermore, this could be symptomatic of the common phenomenon of paper survival in scientific outlets, in our concrete case overrepresenting papers with strong positive effects. This means that the latter would have a higher probability of getting published if they exhibit strong positive effects. However, we leave this investigation for future research, as such an analysis is out of the scope of this paper.

We organized our findings into four sub-sections. First, we shed light on the dependent variables used in IA research; before examining the independent variables found in the retrieved literature. Then, we analyze the respective relationships between independent and dependent variables. Finally, this allows us to construct a conceptually sound research space spanned by the dimensions of independent and dependent variables used in IA research, i.e., design elements for IA user acceptance, which we derived from our systematic literature review.

### Findings on Dependent Variables

The publications at hand adopted a wide dispersion of dependent variables. We identified 213 unique dependent variables (DV). We categorized these 213 DV into three broad categories: Social elements, relational elements, and functional elements.

#### Relational Element (Rapport and Trust)

Researchers have generally examined a plethora of outcomes related to the quality of the social bond between the user and the IA, which is also referred to as *rapport* (Pecune et al., 2018). A third of the studied outcome variables were assigned to this category. Prominently studied variables in this category were the *likeability* of the IA (e.g., Chin & Yi, 2019; Miehle et al., 2018), the degree of *involvement* or *engagement* experienced by users (e.g., Van Es et al., 2002; Vugt et al., 2008), and the *perceived closeness* (Bickmore & Picard, 2005; SeoYoung Lee & Choi, 2017). Additionally, a major outcome category was reflected by user *trust*. As many researchers cited a lack of *trust* as one of the central adoption barriers for AI-based technologies, this sentiment has also been important to *trust* researchers in regard to IAs, making *trust* one of the main variables in the focus of IA research (e.g., Kang & Wei, 2018). However, authors have also investigated trust-related concepts such as *credibility* (e.g., Cowell & Stanney, 2005) or *privacy* perceptions (e.g., Benlian et al., 2019), which we incorporated in this section.

#### Social Elements (Social Presence and Perceived Humanness)

Another important outcome category represented *social elements*, which does not seem surprising since many researchers work on recreating human-IA interactions that are experienced as human-like. Within this category, researchers focused on *perceived humanness* (e.g., Candello et al., 2017) and *social presence* (e.g., Cho, 2019) as the two main outcome variables.

#### Functional Elements (Perceived Ease of Use, Perceived Usefulness, and Subjective Social Norms)

A multitude of authors investigated productivity-related perceptions, which we summarized under the category of *functional elements*. Thereby, prior researchers have looked at *usefulness* (e.g., Qiu & Benbasat, 2010), *ease of use (*e.g., Van Es et al., 2002*)*, and subjective social norms such as *the quality of interaction* (e.g., Ashktorab et al., 2019) and *satisfaction* (e.g., Chaves & Gerosa, 2018).

### Findings on Independent Variables

We identified 390 independent variables (IV) used in IA research. To facilitate the discussion of this high quantity of independent variables, we categorized them into five broader categories. Thereby, our allocation into aggregated dimensions was based on a taxonomic classification of the social cues of IAs introduced by Feine et al. (2019), which we extended based on our coding of selected categories, as some design elements did not fit these categories (i.e., *interaction*). Each category is briefly discussed below.

Within the ***Auditory (3.3%)*** category, the elements that related to *voice qualities* (Schmitt et al., 2021), representing permanent and adjustable characteristics of the voice, were analyzed. In total, these cues were investigated 10 times. For example, Yu et al. (2019) studied the impact of the voice’s gender (female vs. male) on different perceptual outcomes. Although this category hypothetically also included nonlinguistic vocals and sounds, there were no studies in our sample addressing these elements.

Within the ***Interaction (14.5%)*** category, we summarize all design elements that refer to the underlying structural representation of the interaction both in regard to its communication mode and its turn-taking mechanism. Overall, the researchers often studied the choice of *interaction mode.* Moreover, researchers studied the influence of preset answers, which reflects the *degree of freedom* employed in the conversation. The former category was studied 34 times, whereby most researchers compared chat and voice interfaces (Kim et al., 2019). In comparison, the latter category was studied less but was found equally influential for user perceptions (Behera et al., 2021; Diederich et al., 2019).

Design elements in the ***Invisible*** (10.6%) can be divided into four subcategories: Chronemics refers to the role of timing in conversation and is reflected in studies that focused either on the *design of conversation flows* (e.g., Winkler et al., 2019), *system response times* (e.g., Gnewuch et al., 2018), or the *role of synchronicity* (e.g., Park & Sundar, 2015), which in total was studied 11 times. Intelligence refers to elements that express the cleverness of the agent, which was exemplarily studied by Xu et al. (2017). The other two categories, personality (i.e., personality traits) and haptics (i.e., tactile sensations), were comparatively less frequent in the research field. Among personality, e.g., Cafaro et al. (2013) examined the various personality traits.

The ***Visual (34.3%)*** category can be distinguished into four subcategories, which were widely studied (104 times). The most prominently researched variable was *agent appearance* (46 times). The embodiment of the agent gained much attention in our sample (e.g., McBreen & Jack, 2001; Nunamaker et al., 2011). Another studied aspect of the agent’s appearance was gender (e.g., (Pfeuffer et al., 2019; Zhang et al., 2020). Furthermore, *kinesics*, which refers to body movements such as demeanors (e.g., Krämer et al., 2013) and gaze patterns (e.g., Van Es et al., 2002), was addressed 25 times in total*. Computer-mediated communication* (CMC) refers to visual elements that augment or modify written texts and was examined 23 times. Here, the effects of using emojis (e.g., Park & Sundar, 2015), typos (e.g., Westerman et al., 2019), or videos and images (Huber et al., 2018) were researched. The other category, *entrainment* (i.e., the adjustment of visual elements to the user), was studied 20 times in total.

The ***Verbal (37.3%)*** category refers to all IA elements that can be expressed by words, either written or spoken (Antaki, 2008). Within this dimension, the *conversation style*, which refers to how something is being communicated, was the most researched independent variable (53 times). For instance, Mayer et al. (2006) studied the effects of relational strategies. The aspect of *content* captures all elements that relate to the literal meaning of a message and was researched a total of 22 times. For example, Akahori et al. (2019) looked at the effects of self-disclosure. Similar attention was given to *adaptivity* (22 times), which refers to the verbal adaptation of the IA to the users. Within this category, researchers studied the use of contextual information (e.g., Vtyurina et al., 2017), user content (e.g., Schuetzler et al., 2014), or the absence of adaptivity (e.g., Engelhardt et al., 2017).

### Findings on the Relationship between Independent and Dependent Variables

In this section, we summarize our major findings concerning the 49 relationships we coded between 16 IVs and three DVs. At this detailed level, the frequency with which findings were replicated across studies was minimal and did not provide a very coherent or comprehensive picture of IA research. Hence, to study these relationships in a way that would be concise and helpful to researchers, we moved to a higher unit of analysis by reporting the 277 findings using our three categories of DVs and the five categories of IVs. Although precision was reduced when aggregating to the broader categories of DVs, we gained a better overall understanding of the determinants of perceptual and attitudinal outcomes of IAs. Thereby, we also aim to investigate the consistency of the empirical evidence. A detailed table of all included relationships between design Elements (IVs) and DVs can be found in the Appendix of this work. There we specify the IV first and second order constructs, the DV first and second order constructs, and indicate the relationships that could be measured between the IVs and the DVs. In terms of consistency, we looked for variables where at least 60% of the proof was reliable. This minimum threshold was chosen to ensure that more than half of the data yielded the same results. Furthermore, we have to note that findings previously coded with “M”, which referred to a qualitative study, were excluded in the final analysis of the relationships between independent and dependent variables. In the following, we structure our findings along the three DVs and visualize the relationships between the IVs and the DVs in respective figures. As we aim to provide a distinguishable indication on the most reliable results, we created a layered legend. We used the symbol ‘(++)’ to indicate when in more than 70% of investigated IV-DV-relationships, the authors discovered a positively significant relationship. If 51 to 69% of the coded relationships were positively significant, we used a ‘(+)’. Similarly, ‘(− −)’ denotes that in more than 70% of measured relationships between a particular IV and a particular IV were negatively significant, while ‘(−)’ denotes that 51–69% of measured relationships were negatively significant. Clearly, results with greater than 70% consistency are more reliable than those with between 51% and 69% consistency. These cutoff points are determined by the decision rules we used, but since all of the data is in Appendix 1, other researchers are able to re-run studies with different decision rules.

#### Independent Variables on Relational Elements

**Fig. 4:**
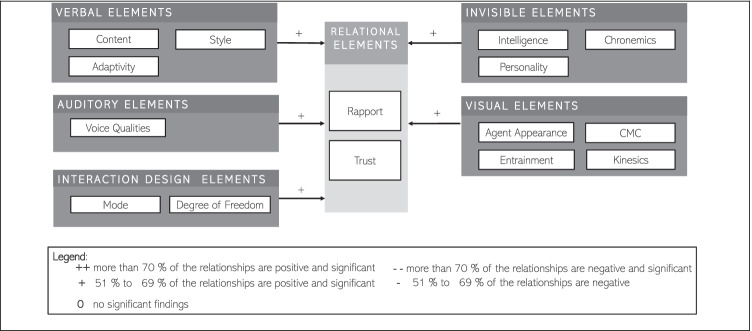
Determinants of relational elements reported by past empirical research on IAs

Figure 4 provides an overview of the relationships between *relational elements* and the five IVs. In this model, 121 findings are synthesized and depicted based on consistency within the subgroups of IVs, providing an answer to the question “*Which determinants of relational elements were reported by past empirical research on IAs*?”

Looking at the results that concern relational elements, we can see that no empirical study has shown clear positive relationships between design elements and relational elements so far. What we do learn from the study is that the nature of IAs has an effect on rapport and trust. Thereby, in the following section, we will go through the first order independent variables for trust and rapport individually in order to have a closer look at the relationships.

Past research offers some evidence that ***verbal design elements*** are determinants of *trust.* Researchers focused their investigation on the variables *style* (9 OBS, e.g., Kang & Wei, 2018), *adaptivity* (8 OBS, Engelhardt et al., 2017), and *content* (5 OBS, e.g., Benlian et al., 2019). The variable *style* produced nearly consistent results. In contrast, the connection between *adaptivity* and *trust* showed mixed results. The variable content, in turn, showed promising results. In this case, three findings were significant, and two more were investigated qualitatively. On the contrary, past research offers little evidence that ***visual design elements*** of intelligent agents are determinants of *trust.* Prior research investigated the variables *agent appearance* (8 OBS, e.g., Nunamaker et al., 2011), *kinesics* (3 OBS, e.g., Elkins & Derrick, 2013), and *proxemics* (1 OBS, Benlian et al., 2019). Similarly, prior research was not able to provide evidence that ***invisible design elements*** of intelligent agents are determinants of *trust.* Nevertheless, past research investigated the variables *personality* (3 OBS, e.g., Nordheim et al., 2019) and *chronemics* (1 OBS, Benlian et al., 2019). Concerning the variables personality and chronemics and their influence on *trust*, no conclusive findings were elaborated. Additionally, there is little evidence that ***auditory design elements*** of intelligent agents are determinants of *trust.* Nevertheless, past research focused on *voice qualities* (3 OBS, e.g., Muralidharan et al., 2014) and has found strong and consistent results. Moreover, prior investigations did not offer any evidence that ***interaction design elements*** are determinants of *trust.* However, the results obtained had a qualitative character. To summarize our findings on the dependent variable *trust,* the most significant and consistent evidence regarding determinants of this outcome dimension was found to be related to the *auditory* design element of *voice qualities*. Additional evidence concerned the three groups of variables coded as *style and content* (*verbal*), *mode* (*interaction*), and *kinesics (visual).* Other variables have not yet been able to show significant evidence in relation to *trust*. However, there are some promising avenues for future research.

When we have a closer look at the dependent variable *rapport*, past research offers evidence that ***verbal design elements*** are an antecedent*.* Researchers investigated the variables *style* (20 observations (OBS), e.g., Clark et al., 2019), *adaptivity* (10 OBS, e.g., Lee et al., 2019), and *content* (4 OBS, e.g., Clark et al., 2019). In this regard, content and entrainment were identified as having a significant impact. For instance, it was shown that eliciting similar interests (Clark, Munteanu, et al., 2019) and the degree of matching or coordination in the word counts of the IA and the user positively influence rapport-building (Pecune et al., 2018). Furthermore, our findings indicate that ***visual design elements*** are determinants of *rapport.* Researchers studied the variables *agent appearance* (19 OBS, e.g., Sproull et al., 1996), *kinesics* (12 OBS, e.g., Krämer et al., 2013), *entrainment* (4 OBS, e.g., Qiu & Benbasat, 2010), and *CMC* (3 OBS, e.g., Westerman et al., 2019). Thereby, *agent appearance* and *CMC* were found to be significant. For instance, enriching the IA’s message by way of typos and capitalization uncovered a significant influence on the social attractiveness of the IA (Westerman et al., 2019). Moreover, including typos and capitalization as manifestations of CMC increased the social attractiveness of the IA (Westerman et al., 2019). In our model, variables also related to ***invisible design elements*** were found to be significant and consistent determinants of *rapport*. Researchers inquired into the variables *intelligence* (6 OBS, e.g., Schuetzler et al., 2019), *chronemics* (2 OBS, e.g., Winkler et al., 2019), and *personality* (1 OBS, Cafaro et al., 2013). In contrast, past research was found to have directed only limited attention to the influence of ***auditory design elements*** on *rapport* between the user and IA. Only one variable (i.e., voice pitch) was studied (Yu et al., 2019), indicating no conclusive evidence. Additionally, our findings indicated some evidence regarding the influence of ***interaction design elements*** as determinants of *rapport* between the user and IA*.* Researchers studied two variables, *mode* (9 OBS, e.g., Miehle et al., 2018) and *degree of freedom* (1 OBS, Jeong et al., 2019)*.* The influence of *mode* was found to be significant. For instance, employing voice-based interfaces increased the users’ self-disclosure towards the IA (Yu et al., 2019). To summarize our findings on the DV *rapport,* the most significant and consistent evidence regarding determinants of this outcome dimension was found to be related to the group of variables coded as *intelligence (invisible).* Other consistent findings were found regarding the variables categorized as *agent appearance* and *CMC (*both *visual), mode (interaction),* and *content* (*verbal).*

#### Independent Variables on Social Elements

**Fig. 5:**
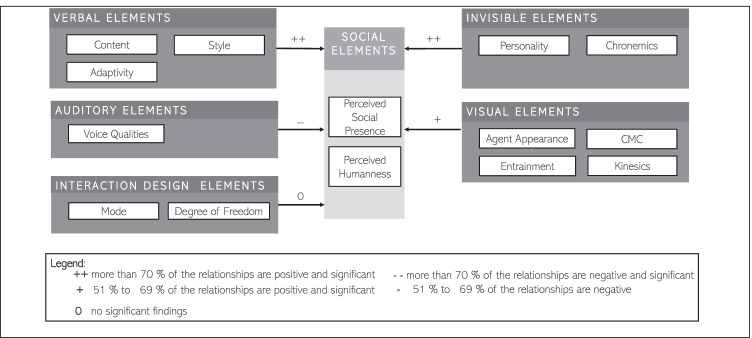
Determinants of social elements reported by past empirical research on IAs

Our findings regarding the outcome dimension of *social elements* are outlined in Fig. 5. In this model, 49 findings were synthesized and depicted based on their consistency within the subgroups of the five second order IVs, providing an answer to the question, “*Which determinants of social elements were reported by past empirical research on IAs*?”

We discovered that the verbal and invisible elements have a strong positive impact on the social elements in this model. Though visual elements have a positive impact, relationships are only positive in 57% of the cases. Surprisingly, auditory elements have so far had a detrimental impact on social elements. There were few significant relationships found between the independent variables mode and degree of freedom, and social elements. In the following section, we will go through the first order independent variables and the findings of the relationship with social elements.

Past research offers some evidence that ***verbal design elements*** of intelligent agents are determinants of *social elements.* Researchers investigated the variables *style* (6 OBS, e.g., Bickmore & Schulman, 2007), *content* (3 OBS, Kobori et al., 2016), and *adaptivity* (3 OBS, Schuetzler et al. 2014). For instance, within the variable *content,* researchers found that small-talk utterances increased the perception of the liveliness of the agent (Kobori et al., 2016). Moreover, in our sample, we found considerable evidence of ***visual design elements*** being determinants of *social elements.* Researchers investigated the variables *CMC* (8 OBS, e.g., Candello et al., 2017), *agent appearance* (7 OBS, e.g., Lee et al., 2019), *kinesics* (3 OBS, Van Es et al., 2002), and *entrainment* (2 OBS, Qiu & Benbasat, 2010). For instance, an IA with a humanoid embodiment was found to be perceived as significantly higher in *social presence* as compared to an IA with no embodiment features. Additionally, our study uncovered considerable evidence suggesting that ***invisible design elements*** are determinants of *social elements.* Researchers investigated the variables *chronemics* (3 OBS, e.g., Gnewuch et al., 2018) and *personality* (2 OBS, e.g., Liao et al., 2018). For instance, dynamic delays in system response time, compared to near-instant responses, were observed to invoke higher perceptions of *social presence* and naturalness of the interaction (Gnewuch et al., 2018). Furthermore, previous research on ***auditory design elements*** identified consistent and significant evidence on *social elements* (4 OBS, Qiu and Benbasat, 2009). Additionally, *Voice qualities* were found to be a significant determinant of *social elements.* For instance, low pitch contour and high flanging increments were found to significantly affect perceptions of humanness (Muralidharan et al., 2014). In addition, past research studying ***interaction design elements*** on *social elements* identified consistent and significant evidence*.* Researchers studied the variables *mode* (4 OBS, Cho, 2019) and *degree of freedom* (3 OBS, Diederich et al., 2019). For example, pre-defined answer options were found to negatively affect perceptions of humanness (Diederich et al., 2019).

To summarize our findings on the DV *social presence and perceived humanness,* the most significant and consistent evidence regarding its determinants was found to be related to the two groups of variables coded as *content* (*verbal*) and *chronemics (invisible).* Other consistent and significant evidence was found regarding the variables *agent appearance* and *CMC (*both *visual), voice qualities (auditory),* and *degree of freedom (interaction).*

#### Independent Variables on Functional Elements

**Fig. 6:**
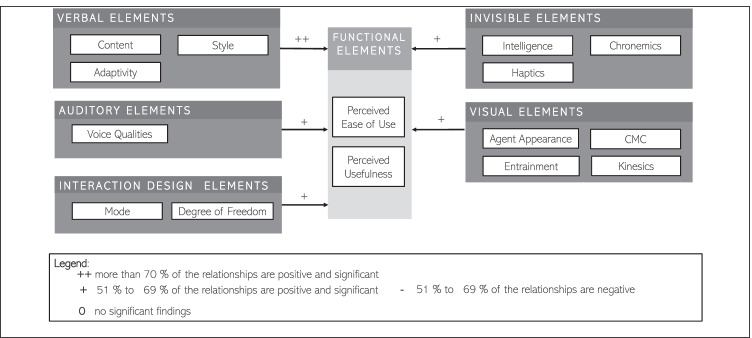
Determinants of functional elements reported by past empirical research on IAs

Our findings regarding the outcome dimension of *functional elements* are outlined in Fig. 6. In this model, 90 findings were synthesized and depicted based on their consistency within the subgroups of the IVs, providing an answer to the question, “Which determinants of *social elements* were reported by past empirical research on IAs?”

We found that only verbal elements had a strong positive relationship to functional elements of IAs. Whilst the other four variables displayed in comparison to verbal elements a weaker relationship, it nevertheless was positive. In the following section, we will go through the first order independent variables and the findings of the relationship with functional elements.

Past research offers no evidence that ***verbal design elements*** are determinants of *functional elements.* Researchers investigated the variables *style* (13 OBS, e.g., Kim et al., 2019), *content* (8 OBS, e.g., Kobori et al., 2016), and *adaptivity* (10 OBS, Engelhardt et al., 2017). The variables *style, adaptivity,* and *content* of the conversation have not yet been able to show evidence that the relationship to *functional elements* is significant. In contrast, we found considerable evidence of ***visual design elements*** being determinants of *functional elements.* Prior research has focused on the variables *CMC* (10 OBS, Westerman et al., 2019), *kinesics* (6 OBS, Cowell & Stanney, 2005), *agent appearance* (5 OBS, e.g., McBreen & Jack, 2001), and *entrainment* (2 OBS, Koulouri et al., 2016). For example, the embodiment of the IA with facial expressions was perceived as more useful, and the users seemed to be more satisfied than with the faceless IA (Sproull et al., 1996).

Users seemed to be more satisfied when the IA had a controlled but normal gaze pattern than when it had a randomized gaze pattern (Van Es et al., 2002). Furthermore, past research offers some evidence that ***invisible design elements*** are determinants of *functional elements.* In our sample of coded findings, researchers focused on the variables *intelligence* (6 OBS, e.g., Xu et al., 2017), *chronemics* (5 OBS, Chaves & Gerosa, 2018), and *haptics* (1 OBS, Kim et al., 2018). For instance, the perceived usefulness was higher when the IA was empowered by deep learning than when it was not (Xu et al., 2017). Further, we found some evidence that ***auditory design elements*** are determinants of *functional elements.* To date, only the influence of *voice qualities* has been investigated, but no significant evidence for other design elements could be found (Tian et al., 2017). *Voice qualities*, i.e., the distinctive characteristics between acted and natural speech, did affect how well the IA recognized the users’ emotions. Additionally, past research offers some evidence that ***interaction design elements*** are determinants of *functional elements.* Prior researchers have found significant evidence when looking at the variable *mode* (15 OBS, Miehle et al., 2018). Concerning *degree of freedom* (9 OBS, Mu & Sarkar, 2019), for example, Akahori et al. (2019) were able to show that the main effects of the number of agents had a significant influence on understandability. To summarize our findings on the dependent variable *usefulness,* the most significant and consistent evidence regarding determinants of this outcome dimension was related to the three groups of variables coded as *intelligence (invisible), agent appearance,* and *kinesics* (both *visual*)*.* Further consistent and significant evidence was found regarding the variables *CMC (visual), degree of freedom,* and *mode (*both *interaction).*

#### Spanning the Research Space of Designing IAs for User Acceptance

Based on our literature analysis presented above, we are able to provide researchers with a conceptually sound research space encompassing empirical HCI and IS research on the design elements for IA user acceptance. The research space is spanned by the independent (IVs) and dependent variables (DVs), which we derived from the systematic literature review. Aiming for conciseness and usability of the research space, we aggregate the IVs to interaction, visual, verbal, auditory, and invisible design elements. Furthermore, we view the DVs on the level of abstraction of the HCI framework – i.e., relational, social, and functional elements. Table 3 visualizes the research space of designing IAs for user acceptance and provides a summary of the progress in the various combinations of design elements and attitudinal and perceptional outcomes in the realm of IAs. Each combination can be deemed to present a research avenue that has been explored at different levels of intensity.

**Table.3:**
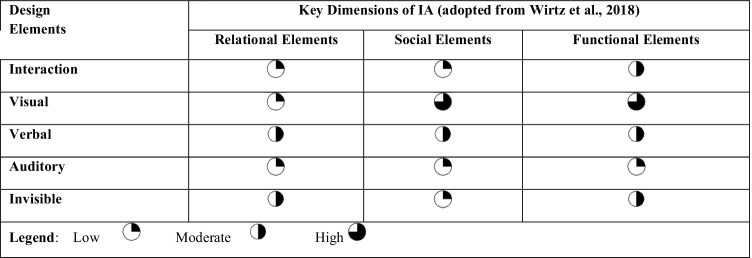
Research space and summary of research progress

We use Harvey Balls to indicate how developed a particular research avenue in the field of designing for IA user acceptance is regarding the number and content of the respective contributions. Avenues in which only a few items were found or the contributions were represented only a first attempt at research (i.e., only qualitatively) were classified as *“low”*. Fields with some contributions or mixed results were classified as *“moderate”*. Fields with several contributions and consistent findings were classified as *“high”*. In connection with the descriptive and thematic findings, this assessment provides the opportunity to identify avenues for further research. Thus, in each thematic discussion, we were facilitated to localize and describe fruitful research opportunities according to the framework of Müller-Bloch and Kranz (2015). Based on this, we present an extensive research agenda along the dimensions of the derived research space of designing for IA user acceptance in the next section. This shall be useful to both researchers and practitioners, thus complementing the past and present knowledge on designing for IA user acceptance with potential pathways into the future of IAs.

## Research Agenda

In the light of the findings presented in Section 5, several research gaps are identified in relation to the study of the design of IAs. This section aims to develop a research agenda for guiding future research on designing for IA user acceptance by gaining a deeper understanding of the underlying assumptions and highlighting areas where there is a significant lack of knowledge in a structured and transparent manner. We position the research agenda research space that we have spanned in this review by linking outcomes of IAs to categories of design elements. The goal is not to present an exhaustive list of potential research streams but rather to showcase some critical gaps in our understanding of how the design of IAs is influencing how users interact with and accept them.

### Research Avenues for Designing Relational Elements for IA User Acceptance

With regard to the relational elements of agent acceptance, prior publications provided insights into design elements that contribute to forming a social bond with the agent. In total, we found 121 relationships involving *relational elements* within 57 unique studies, which testifies to the high research interest in this aspect of user interaction with IAs. Thereby, several relationships showed consistent and robust evidence of a positive effect on *relational elements* such as rapport or trust. Overall, the relationships investigated were positive, but no consistent findings were discovered between relational elements and any of the five design elements. As a result, we believe that additional research on how verbal, visual, auditory, interaction, and invisible design elements affect user acceptance of IAs is necessary.

We structure the potentially fruitful future research directions for designing relational elements for IA user acceptance along the five categories of design elements investigated in this study:

#### Interaction

Understanding how *relational elements* emerge between the user and the IA in the context of different interaction modes has been the focus of several studies. In our review, we extracted a multitude of findings related to the effects of the interaction mode on *relational elements*. For instance, D’Mello et al. (2010) investigated how the mode of interaction (text-based vs. voice-based) affects the dynamics between students and an IA tutor, but the limited sample of the study moderated the explanatory power. Thus, we argue that exploring IA interaction modes and their influence on *relational elements* represents an insightful direction for future research. Since researchers have found that people react to IAs in ways that are close to how they would respond to humans in a number of studies in dyadic interaction, future research should have a closer look at how interaction can be designed when IAs are part of a team (i.e., Elshan et al., 2022; Elshan & Ebel, 2020) and how this would influence the acceptance on individual user, team, and potentially organizational levels.

#### Verbal

Across different studies, 56 findings were concerned with investigating the influence of the IA’s verbal style on dynamics between the user and the IA. Adaptivity of IAs, as shown by Lee et al. (2019), in particular, is likely to become much more important in the coming years as technology and its ability to adapt to user content develop. This will also allow us to create more personalized and individualized interactions with the IAs. In this realm, the question arises which application domains will benefit from this adaptivity and which would benefit from a non-adaptive IA. Furthermore, as IAs are becoming prevalent actors in our daily lives, the content which is being communicated from IAs to humans might have effects on user acceptance. This bears a high potential for future research examining, for example, when users feel annoyed by the content the IA is transporting. In the future, not only the aspect of rapport but also the aspect of trust can be investigated further. Here, researchers might want to investigate when and why users are trusting IAs, and probably, even more, important which factors lead to distrust causing large-scale harm such as brand damage or bad word-of-mouth. Against the backdrop of the rising interest in explainable artificial intelligence, future studies could focus on how IAs can communicate in a transparent way to serve as a facilitator of technological innovation. Therefore, we identify *relational elements* between the user and the IA in the context of different IA verbal styles as a highly worthwhile avenue for HCI and IS researchers.

#### Invisible

Examining invisible design elements of IAs, notably, the IA’s intelligence seems to afford a positive user evaluation of relationship to the agent (e.g., Xu et al., 2017). However, there are inconsistent findings, as shown in this review (e.g., Winkler et al. (2019) vs. Pecune et al. (2018) researching the relationship between *chronemics* and *rapport*), which prompts further research. Future research should examine the effect of an IA outperforming a human in regard to intelligence. Certainly, the design element of intelligence is highly task- and context-related, the cleverness might lead to a worse rapport towards the agent (i.e., Schuetzler et al., 2019). Concerning other perspectives of invisible design elements, the reasoning of Nordheim et al. (2019) indicates that attributing personality to IAs shall positively affect perceptions of trustworthiness. However, the papers in our sample showed inconclusive evidence regarding the effects of agent personality. Hence, we propose that future research further explores the attribution of personality dimensions (i.e., Big Five; introversion vs. extroversion) in IAs and their effects on *relational elements*.

#### Visual

In general, agent appearance has been explored relatively often regarding user trust (e.g., Nunamaker et al., 2011) and rapport. However, our findings only heralded mixed results. It seems that *agent appearance* is vital in some contexts but not in others. Additionally, we have little to no prior evidence on the effect of CMC has on relational elements. We know very little about when it makes sense to use visual elements in the context of CMC at all and when it is appropriate to do so to build up a relationship between users and IAs. Hence, there is a need to explore the effect of appearance design from a more nuanced perspective taking into account the respective context or task.

#### Auditory

Overall, we can state that there has been little to no prior research on auditory elements affecting the users’ acceptance. Furthermore, most of the prior research has focused mostly on the effects of the voice pitch on trust or rapport (e.g., Elkins & Derrick, 2013). Since most of the time, auditory aspects such as the voice quality are predefined by the platform, the IAs is run on (i.e., Apple’s Siri is by default female). Therefore, moving beyond default voice qualities could be a way forward for future research.

### Research Avenues for Designing Social Elements for IA User Acceptance

Regarding the social elements of agent adoption*,* we identified 49 findings within 29 unique studies. Thereby, to no surprise, the design elements that represented social cues of IAs, especially, showed a strong and consistent effect on *social presence and perceived humanness*. However, the findings were especially related to appearance were not as consistent as one would have expected, especially since agent appearance is a well-researched area when considering early research on other agents (Nowak & Biocca, 2003). Thus, future research should incorporate a more fine-grained and configurational view on these design elements since we suspect that the interrelationship could be key in understanding *social presence* with IAs. Hence, we propose the following prospects for future research:

#### Interaction

Initial findings suggest that speaking versus talking with an interface can influence perceptions of social presence (Hess et al., 2009), such that generally voice-based interfaces are perceived as being more human-like. However, there is a lack of contextual perspectives that delimitate the boundary conditions of the effect of modality on social presence. Moreover, it is not clear if and under which conditions these perceptions translate into positive downstream consequences for firms, such as increased usage or increased disclosure behavior.

#### Visual

In general, there has been a sizeable amount of research that shows that visual design elements in specific amounting to avatars are paramount for increasing humanness in IAs. However, future research should provide a more in-depth understanding of which appearance elements positively affect social presence and to what degree. Arguing from an “uncanny valley” perspective (Mori et al., 2012), specific elements of agent appearance could be related more consistently to leveraging *social presence* perceptions than others, especially anthropomorphic design elements (Pfeuffer et al., 2019). In connection with the previously mentioned first avenues for future research, we suggest that agent appearance from a visual perspective, as well as other elements leveraging *social presence,* should not be treated in isolation but rather carefully considered in a configurational view, for instance, with other aspects such as personality of the agent, which, so far, has been neglected in research. For example, Amazon’s Alexa has a very minimalistic appearance but a high degree of *social presence* through other elements fostering *social presence* (Purington et al., 2017).

#### Voice

Initial research suggests that voice characteristics are an important determinant of perceptions of social presence and humanness when interacting with voice-based IAs. While our review shows that different characteristics related to the quality of the voice are an important driver of perception of humanness, a multitude of characteristics that are known to affect personality perceptions in human-to-human conversations such as pitch or pace (Chang et al., 2018) have not been investigated in the context of voice-based IAs. Moreover, it is not clear under which conditions specific voice features are perceived as humanlike, which poses novel research opportunities.

#### Invisible

Initial research shows that invisible cues such as chronemics can be instrumental for users perceiving an IA as being more human. Most of this research has focused on the role of typing indices to mimic human behavior (Gnewuch et al., 2018). One important factor in human-agent interaction that has been studied less is the locus of control. In this regard, it is not clear in which contexts and tasks the role of leadership in the interaction may affect perceptions of social presence. For instance, while in some cases, such as social robots, it might make sense to give control to the user from a practical perspective. However, this might decrease the user’s perception of humanness as the agent is passive. Thus, locus of control is a promising opportunity for IS and HCI researchers.

#### Verbal

Past research offers some evidence that the way the IA talks to the user is a distinguishing factor for perceiving an agent as humanlike or not. Especially, the notion of small-talk was identified as promoting perceptions of humanness (Kobori et al., 2016). However, there is a lack of rigorous experimental research that investigates the effect of conversation design on social presence and its downstream consequences. In specific, there are few concrete guidelines that help researchers and practitioners to design conversation flows that they perceive as authentic by the user. Moreover, the question arises if there are trade-offs between efficient dialogue-structures, for instance, by using a task-oriented versus relational-oriented conversation style and how these trade-offs can be balanced against each other depending on the context.

### Research Avenues for Designing Functional Elements for IA User Acceptance

In the analyzed literature, manifold insights into how design elements contribute to creating *functional elements* for the user were gained. In this research stream, we identified 90 findings involving *functional elements* within 41 unique studies. Those with characteristics, especially regarding utility, were concerned with the accessibility or functionality of the interaction. Thus, we found strong evidence for the effects of *degree of freedom* (interaction), *intelligence* (invisible), *agent appearance*, and *kinesics* (visual). Moreover, we found consistent evidence that *adaptivity* (verbal), *CMC* (visual), and *mode* (interaction) may be positively related to *functional elements*; however, these results should be corroborated by further studies and replicated in different contexts. In general, the results regarding *utility* perceptions are already quite profound. However, we see merit, especially in the following research directions:

#### Verbal

Based on theoretical reasoning and qualitative data, several researchers highlighted that *adaptivity* might afford high potential for creating *functional elements,* as users expect personalized content. However, other researchers argued that standardized content might contribute to ease of use (Chin & Yi, 2019). Thus, we propose to investigate the effect of IA *adaptivity* on *utility* in different contexts, especially focusing on the tradeoffs between standardization and personalization.

#### Interaction

Researching the *functional elements* of interaction with IA is undoubtedly one of the most common side effects of research in the field of IA. However, very few have been concerned with merely investigating the effects of the chosen interaction *mode* or the *degree of freedom* on *functional elements*. As more people of various ages and backgrounds access and interact with an IA, the interaction must be tailored to the specific user groups. Thus, in the future, it will be investigated whether the degree of freedom should be adapted for different user groups, such as older people so that they perceive ease of use.

#### Visual

Our findings showed that there has already been some prior work in regard to the effects of visual design elements on functional elements, such as the *ease of use*. So far, studies have shown that users seem to be more satisfied when the IA had a controlled but normal gaze pattern (Van Es et al., 2002). Having this in mind, a possible pathway for future research might be to examine the adjustment of visual elements to the user’s input. Furthermore, future studies could have a look at the design of visual elements affecting the functional elements when IAs are integrated into a group of people. Therefore, prior insights of the effect of agents’ appearance on functional elements can be investigated in the context of teams.

#### Invisible

Current research did hardly investigate how the design of personality affects the ease of use or perceived usefulness of IAs. Thereby future research may focus on this aspect and investigate specific configurations of the IA’s personality. Nevertheless, first, a structured investigation of the possible contextual factors of an interaction is necessary. Afterward, different application domains and specific use cases of IAs are worthwhile streams for future research. For instance, an empathetic IA could have a positive effect on the ease of use of chronic ill humans using IAs as a coach.

#### Auditory

So far, we have very little insight into when the design of auditory elements leads to a positive or negative effect on functional elements like perceived usefulness of the IA**.** With the increasing implementation of voice-based IAs in a variety of domains such as e-commerce or banking, further investigations are necessary in order to determine what auditory elements have positive or negative effects on the user’s acceptance of the IA.

Moreover, we have identified some overarching research opportunities based on an overall positioning of the reviewed literature. According to Li and Zhang (2005), HCI is concerned with the interaction between an IS and a user. This interaction is shaped by the characteristics of the system, the user, and the task context. Interaction results can include perceptions, attitudes, intentions, and behaviors (Li & Zhang, 2005). In the scope of reviewing the literature, we found that task context (i.e., support, assistance, coaching function) has rarely been implicated in the research design.

Moreover, user characteristics have rarely been considered in the research model beyond being a control variable. Here, we see an important research gap, as both task and user characteristics may dramatically influence the effect of IA design on user perceptions. Furthermore, the discipline is in dire need of an investigation into the influence of these interfaces and their features on behaviors in real-life settings.

## Conclusion

The holistic evaluation of the empirical academic literature regarding user interaction with IAs is crucial in uncovering potential research avenues for shaping future empirical IA research. For this purpose, we conducted a systematic literature review to study which design elements had a significant influence on design outcomes. Following Jeyaraj et al. (2006), we identified, coded, validated, and analyzed quantitative and qualitative empirical findings on user interaction with IAs. We, therefore, analyzed the 107 identified research papers and systematically identified existing knowledge as well as future research needs. By considering the three major outcomes, social elements, functional elements, and relational elements, we were able to identify a set of variables that takes the variables within these outcomes into account. More precisely, we identified a set of 389 relationships that were examined in the context of IAs. Based on our literature analysis presented above, we are able to provide researchers with a conceptually sound research space encompassing empirical HCI and IS research on the design elements for IA user acceptance. The research space is spanned by the independent (IVs) and dependent variables (DVs), which we derived from the systematic literature review, and contains the 389 coded relationships between IVs and DVs. Based on this, we present an extensive research agenda along the dimensions of the derived research space of designing for IA user acceptance. This shall be useful to both researchers and practitioners, thus complementing the past and present knowledge on designing for IA user acceptance with potential pathways into the future of IAs.

Despite us following established guidelines and attempting to rigorously analyze the identified empirical literature on user interaction with IAs, this SLR has several limitations that should be considered. Despite due diligence, the scope might not be fully exhaustive, and our search strategy may have missed relevant publications. Nevertheless, we aimed at capturing a broad and representative spectrum of research on user interaction with IAs by employing a journal- and proceedings-based search. Second, the indicated relationships between the design elements and the user outcomes are based on our interpretation of prior empirical research.

Furthermore, the number of findings ultimately coded and included in our dataset was limited. Thus, it is not our intention to suggest any kind of causality between the design elements and user outcomes. By applying the method introduced by Jeyaraj et al. (2006), it was our objective to elucidate the variables studied and offer a conceptual structuring of the empirical findings on design elements and their influence on outcomes. Fourth, bias within the results was visible, which consisted of a strong overrepresentation of positive effects, probably rooted in paper survival bias. Finally, the resulting research agenda imposes further limitations. Even though we intend to provide a rich basis future research can build upon, the proposed research agenda cannot be regarded as complete and is thus extendible by design. Here, researchers can extend our work by posing additional research questions and proposing further research streams structured within the research space we span in this paper. Doing so will provide researchers with further means of both explaining and informing the design of useful IAs for practice.

### Conflict of Interest and Acknowledgments

E.E and N.Z. contributed equally. The authors have no conflicts of interest to declare that are relevant to the content of this article. We thank the Swiss National Science Foundation for funding parts of this research (100013_192718). The fourth author acknowledges funding from the Basic Research Fund (GFF) of the University of St. Gallen.

## Appendix

Table of all included relationships between design elements and dependent variables

**Table:**

Source	ID	IV second order	IV first order	Relationship	DV first order	DV second order
Sproull et al. (1996)	105_1	Visual	Agent appearance	Positive effect on	Rapport	Relational elements
Sproull et al. (1996)	105_2	Visual	Agent appearance	Positive effect on	Usefulness	Functional elements
Sproull et al. (1996)	105_3	Visual	Agent appearance	Positive effect on	Rapport	Relational elements
McBreen et al. (2001)	69_10	Visual	Agent appearance	Positive effect on	Social presence	Social elements
McBreen et al. (2001)	69_11	Visual	Agent appearance	Positive effect on	Social presence	Social elements
McBreen et al. (2001)	69_12	Verbal	Style	No effect on	Trust	Relational elements
McBreen et al. (2001)	69_13	Verbal	Style	Positive effect on	Rapport	Relational elements
McBreen et al. (2001)	69_14	Visual	CMC	Positive effect on	Usefulness	Functional elements
McBreen et al. (2001)	69_15	Visual	CMC	No effect on	Usefulness	Functional elements
McBreen et al. (2001)	69_16	Visual	Agent appearance	Positive effect on	Rapport	Relational elements
McBreen et al. (2001)	69_17	Visual	CMC	Positive effect on	Rapport	Relational elements
McBreen et al. (2001)	69_18	Visual	Kinesics	Positive effect on	Rapport	Relational elements
McBreen et al. (2001)	69_19	Visual	Kinesics	No effect on	Rapport	Relational elements
McBreen et al. (2001)	69_2	Visual	Agent appearance	Positive effect on	Rapport	Relational elements
McBreen et al. (2001)	69_3	Visual	Agent appearance	No effect on	Rapport	Relational elements
McBreen et al. (2001)	69_4	Visual	Agent appearance	Positive effect on	Usefulness	Functional elements
McBreen et al. (2001)	69_5	Visual	Agent appearance	Positive effect on	Usefulness	Functional elements
McBreen et al. (2001)	69_7	Visual	Agent appearance	Positive effect on	Rapport	Relational elements
McBreen et al. (2001)	69_8	Visual	Agent appearance	Positive effect on	Rapport	Relational elements
Van Es et al. (2002)	30_1	Visual	Kinesics	Positive effect on	Usefulness	Functional elements
Van Es et al. (2002)	30_2	Visual	Kinesics	Positive effect on	Usefulness	Functional elements
Van Es et al. (2002)	30_3	Visual	Kinesics	Positive effect on	Rapport	Relational elements
Van Es et al. (2002)	30_4	Visual	Kinesics	Positive effect on	Perceived humanness	Social elements
Van Es et al. (2002)	30_5	Visual	Kinesics	Positive effect on	Social presence	Social elements
Van Es et al. (2002)	30_6	Visual	Kinesics	No effect on	Social presence	Social elements
Van Es et al. (2002)	30_7	Visual	Kinesics	No effect on	Usefulness	Functional elements
Bickmore et al. (2004)	35_1	Verbal	Style	Positive effect on	Rapport	Relational elements
Bickmore et al. (2004)	35_2	Verbal	Style	Positive effect on	Rapport	Relational elements
Bickmore et al. (2004)	35_3	Verbal	Style	Positive effect on	Trust	Relational elements
Bickmore et al. (2004)	35_4	Verbal	Style	Positive effect on	Trust	Relational elements
Bickmore et al. (2004)	35_6	Verbal	Style	Positive effect on	Rapport	Relational elements
Bickmore and Picard (2005)	47_1	Verbal	Style	Positive effect on	Rapport	Relational elements
Bickmore and Picard (2005)	47_2	Verbal	Style	Positive effect on	Rapport	Relational elements
Bickmore and Picard (2005)	47_3	Verbal	Style	Positive effect on	Usefulness	Functional elements
Berry et al. (2005)	53_1	Interaction	Mode	Positive effect on	Usefulness	Functional elements
Berry et al. (2005)	53_2	Interaction	Mode	Positive effect on	Trust	Relational elements
Berry et al. (2005)	53_3	Interaction	Mode	Positive effect on	Usefulness	Functional elements
Berry et al. (2005)	53_4	Interaction	Mode	Positive effect on	Rapport	Relational elements
Berry et al. (2005)	53_5	Interaction	Mode	Positive effect on	Usefulness	Functional elements
Berry et al. (2005)	53_6	Verbal	Style	Positive effect on	Usefulness	Functional elements
Berry et al. (2005)	53_7	Verbal	Style	Positive effect on	Trust	Relational elements
Berry et al. (2005)	53_8	Verbal	Style	Positive effect on	Trust	Relational elements
Berry et al. (2005)	53_9	Verbal	Style	Positive effect on	Usefulness	Functional elements
Cowell et al. (2005)	54_1	Visual	Kinesics	Positive effect on	Trust	Relational elements
Cowell et al. (2005)	54_2	Visual	Kinesics	Negative effect on	Usefulness	Functional elements
Cowell et al. (2005)	54_3	Visual	Kinesics	Negative effect on	Usefulness	Functional elements
Cowell et al. (2005)	54_4	Visual	Kinesics	Positive effect on	Usefulness	Functional elements
Bickmore and Mauer (2006)	36_1	Visual	Agent appearance	Positive effect on	Rapport	Relational elements
Bickmore and Mauer (2006)	36_2	Visual	Agent appearance	Positive effect on	Perceived humanness	Social elements
Bickmore and Mauer (2006)	36_3	Visual	Agent appearance	No effect on	Usefulness	Functional elements
Bickmore and Mauer (2006)	36_4	Visual	Agent appearance	Positive effect on	Rapport	Relational elements
Bickmore and Mauer (2006)	36_5	Visual	Agent appearance	Positive effect on	Trust	Relational elements
Bickmore and Mauer (2006)	36_6	Visual	Agent appearance	Positive effect on	Trust	Relational elements
Mayer et al. (2006)	52_1	Verbal	Style	Negative effect on	Rapport	Relational elements
Mayer et al. (2006)	52_2	Verbal	Style	Negative effect on	Rapport	Relational elements
Bickmore and Schulman (2007)	34_1	Verbal	Style	Positive effect on	Social presence	Social elements
Bickmore and Schulman (2007)	34_2	Verbal	Style	No effect on	Social presence	Social elements
Bickmore and Schulman (2007)	34_3	Verbal	Style	No effect on	Social presence	Social elements
Bickmore and Schulman (2007)	34_4	Verbal	Style	No effect on	Social presence	Social elements
Bickmore and Schulman (2007)	34_5	Verbal	Style	Positive effect on	Usefulness	Functional elements
Bickmore and Schulman (2007)	34_7	Verbal	Style	No effect on	Rapport	Relational elements
Bickmore and Schulman (2007)	34_8	Verbal	Style	No effect on	Rapport	Relational elements
Schumaker et al. (2007)	45_1	Invisible	Intelligence	Negative effect on	Usefulness	Functional elements
Schumaker et al. (2007)	45_2	Invisible	Intelligence	Negative effect on	Usefulness	Functional elements
Le Bigot et al. (2007)	50_1	Interaction	Mode	Positive effect on	Rapport	Relational elements
Le Bigot et al. (2007)	50_2	Interaction	Mode	Positive effect on	Rapport	Relational elements
Le Bigot et al. (2007)	50_3	Interaction	Mode	Positive effect on	Rapport	Relational elements
Qiu et al. (2009)	111_1	Visual	Agent appearance	Positive effect on	Social presence	Social elements
Qiu et al. (2009)	111_2	Interaction	Mode	Positive effect on	Social presence	Social elements
Qiu et al. (2009)	111_3	Auditory	Voice qualities	Positive effect on	Social presence	Social elements
Qiu et al. (2009)	111_4	Interaction	Mode	No effect on	Social presence	Social elements
Vugt et al. (2008)	102_4	Visual	Entrainment	No effect on	Rapport	Relational elements
Vugt et al. (2008)	102_6	Visual	Entrainment	No effect on	Rapport	Relational elements
D’Mello et al. (2010)	104_4	Interaction	Mode	Positive effect on	Usefulness	Functional elements
D’Mello et al. (2010)	104_5	Interaction	Mode	Negative effect on	Usefulness	Functional elements
D’Mello et al. (2010)	104_6	Interaction	Mode	No effect on	Rapport	Relational elements
D’Mello et al. (2010)	104_7	Interaction	Mode	No effect on	Usefulness	Functional elements
Qiu et al. (2010)	112_1	Visual	Entrainment	Positive effect on	Social presence	Social elements
Qiu et al. (2010)	112_2	Visual	Entrainment	Positive effect on	Rapport	Relational elements
Qiu et al. (2010)	112_3	Visual	Entrainment	Positive effect on	Usefulness	Functional elements
Qiu et al. (2010)	112_4	Visual	Entrainment	No effect on	Social presence	Social elements
Qiu et al. (2010)	112_5	Visual	Entrainment	No effect on	Rapport	Relational elements
Qiu et al. (2010)	112_6	Visual	Entrainment	No effect on	Usefulness	Functional elements
Nunamaker et al. (2011)	1_1	Visual	Agent appearance	Positive effect on	Trust	Relational elements
Nunamaker et al. (2011)	1_2	Visual	Agent appearance	Positive effect on	Rapport	Relational elements
Nunamaker et al. (2011)	1_3	Visual	Agent appearance	Positive effect on	Trust	Relational elements
Nunamaker et al. (2011)	1_4	Visual	Agent appearance	Positive effect on	Rapport	Relational elements
Nunamaker et al. (2011)	1_5	Visual	Kinesics	Positive effect on	Rapport	Relational elements
Nunamaker et al. (2011)	1_6	Visual	Kinesics	No effect on	Trust	Relational elements
Nunamaker et al. (2011)	1_7	Visual	Kinesics	No effect on	Rapport	Relational elements
Nunamaker et al. (2011)	1_8	Visual	Kinesics	Positive effect on	Rapport	Relational elements
Krämer et al. (2013)	51_1	Visual	Kinesics	No effect on	Rapport	Relational elements
Krämer et al. (2013)	51_2	Visual	Kinesics	No effect on	Rapport	Relational elements
Elkins et al. (2013)	76_1	Visual	Kinesics	Positive effect on	Trust	Relational elements
Elkins et al. (2013)	76_2	Visual	Agent appearance	No effect on	Trust	Relational elements
Elkins et al. (2013)	76_3	Auditory	Voice qualities	Negative effect on	Trust	Relational elements
Cafaro et al. (2013)	88_1	Visual	Kinesics	Positive effect on	Rapport	Relational elements
Cafaro et al. (2013)	88_3	Visual	Kinesics	Positive effect on	Rapport	Relational elements
Cafaro et al. (2013)	88_4	Invisible	Personality	No effect on	Rapport	Relational elements
Schuetzler et al. (2014)	110_1	Verbal	Adaptivity	Positive effect on	Perceived humanness	Social elements
Schuetzler et al. (2014)	110_2	Verbal	Adaptivity	Positive effect on	Rapport	Relational elements
Muralidharan et al. (2014)	72_1	Auditory	Voice qualities	Negative effect on	Trust	Relational elements
Muralidharan et al. (2014)	72_2	Auditory	Voice qualities	Negative effect on	Perceived humanness	Social elements
Muralidharan et al. (2014)	72_3	Auditory	Voice qualities	Negative effect on	Trust	Relational elements
Muralidharan et al. (2014)	72_4	Auditory	Voice qualities	Negative effect on	Perceived humanness	Social elements
Lucas et al. (2014)	79_1	Invisible	Intelligence	Negative effect on	Trust	Relational elements
Lucas et al. (2014)	79_2	Invisible	Intelligence	Positive effect on	Trust	Relational elements
Lucas et al. (2014)	79_3	Invisible	Intelligence	Positive effect on	Trust	Relational elements
Strait et al. (2015)	37_1	Visual	Agent appearance	Positive effect on	Perceived humanness	Social elements
Strait et al. (2015)	37_2	Visual	Agent appearance	Positive effect on	Trust	Relational elements
Park et al. (2015)	77_1	Invisible	Chronemics	Positive effect on	Social presence	Social elements
Park et al. (2015)	77_3	Invisible	Chronemics	Positive effect on	Usefulness	Functional elements
Park et al. (2015)	77_4	Visual	CMC	Positive effect on	Social presence	Social elements
Park et al. (2015)	77_6	Visual	CMC	Positive effect on	Usefulness	Functional elements
Terada et al. (2015)	89_2	Visual	Agent appearance	Positive effect on	Rapport	Relational elements
Terada et al. (2015)	89_3	Visual	Agent appearance	No effect on	Rapport	Relational elements
Koulouri et al. (2016)	103_1	Interaction	Mode	Negative effect on	Usefulness	Functional elements
Koulouri et al. (2016)	103_2	Verbal	Adaptivity	Positive effect on	Usefulness	Functional elements
Koulouri et al. (2016)	103_3	Verbal	Adaptivity	Negative effect on	Usefulness	Functional elements
Koulouri et al. (2016)	103_4	Verbal	Adaptivity	Positive effect on	Usefulness	Functional elements
Cafaro et al. (2016)	106_1	Visual	Kinesics	Positive effect on	Rapport	Relational elements
Cafaro et al. (2016)	106_2	Visual	Kinesics	Positive effect on	Rapport	Relational elements
Wang et al. (2016)	113_1	Verbal	Content	Positive effect on	Trust	Relational elements
Wang et al. (2016)	113_2	Visual	Agent appearance	Positive effect on	Trust	Relational elements
Luger and Sellen (2016)	12_1	Interaction	Mode	Negative effect on	Trust	Relational elements
Luger and Sellen (2016)	12_2	Interaction	Mode	Positive effect on	Usefulness	Functional elements
Shamekhi et al. (2016)	87_1	Verbal	Adaptivity	No effect on	Usefulness	Functional elements
Shamekhi et al. (2016)	87_3	Verbal	Adaptivity	Positive effect on	Rapport	Relational elements
Shamekhi et al. (2016)	87_4	Verbal	Adaptivity	No effect on	Trust	Relational elements
Shamekhi et al. (2016)	87_5	Verbal	Adaptivity	No effect on	Social presence	Social elements
Shamekhi et al. (2016)	87_6	Verbal	Adaptivity	No effect on	Social presence	Social elements
Shamekhi et al. (2016)	87_7	Verbal	Adaptivity	No effect on	Trust	Relational elements
Shamekhi et al. (2016)	87_8	Verbal	Adaptivity	No effect on	Rapport	Relational elements
Kobori et al. (2016)	91_1	Verbal	Content	Positive effect on	Usefulness	Functional elements
Kobori et al. (2016)	91_2	Verbal	Content	Positive effect on	Rapport	Relational elements
Kobori et al. (2016)	91_3	Verbal	Content	Positive effect on	Social presence	Social elements
Kobori et al. (2016)	91_5	Verbal	Content	Positive effect on	Social presence	Social elements
Kobori et al. (2016)	91_6	Verbal	Content	Negative effect on	Social presence	Social elements
Kobori et al. (2016)	91_7	Verbal	Content	No effect on	Usefulness	Functional elements
Wuenderlich et al. (2017)	2_3	Verbal	Style	Positive effect on	Rapport	Relational elements
Xu et al. (2017)	25_1	Invisible	Intelligence	Positive effect on	Rapport	Relational elements
Xu et al. (2017)	25_2	Invisible	Intelligence	Positive effect on	Rapport	Relational elements
Xu et al. (2017)	25_3	Invisible	Intelligence	Positive effect on	Usefulness	Functional elements
Xu et al. (2017)	25_4	Invisible	Intelligence	Positive effect on	Rapport	Relational elements
Xu et al. (2017)	25_5	Invisible	Intelligence	Positive effect on	Rapport	Relational elements
Xu et al. (2017)	25_6	Invisible	Intelligence	Positive effect on	Usefulness	Functional elements
Lee et al. (2017)	49_4	Verbal	Content	Positive effect on	Rapport	Relational elements
Lee et al. (2017)	49_5	Verbal	Content	Positive effect on	Trust	Relational elements
Lee et al. (2017)	49_6	Verbal	Content	Positive effect on	Rapport	Relational elements
Tian et al. (2017)	66_1	Auditory	Voice qualities	Positive effect on	Usefulness	Functional elements
Tian et al. (2017)	66_2	Auditory	Voice qualities	Positive effect on	Usefulness	Functional elements
Vtyurina et al. (2017)	7_1	Verbal	Adaptivity	Positive effect on	Usefulness	Functional elements
Vtyurina et al. (2017)	7_2	Verbal	Adaptivity	Positive effect on	Usefulness	Functional elements
Vtyurina et al. (2017)	7_3	Verbal	Content	Positive effect on	Trust	Relational elements
Engelhardt et al. (2017)	71_1	Verbal	Adaptivity	Effect on	Rapport	Relational elements
Engelhardt et al. (2017)	71_2	Verbal	Adaptivity	Effect on	Rapport	Relational elements
Engelhardt et al. (2017)	71_3	Verbal	Adaptivity	Effect on	Rapport	Relational elements
Engelhardt et al. (2017)	71_4	Verbal	Adaptivity	Effect on	Trust	Relational elements
Engelhardt et al. (2017)	71_5	Verbal	Adaptivity	Effect on	Trust	Relational elements
Candello et al. (2017)	9_1	Visual	CMC	Negative effect on	Perceived humanness	Social elements
Candello et al. (2017)	9_2	Visual	CMC	Negative effect on	Perceived humanness	Social elements
Liao et al. (2018)	14_2	Invisible	Personality	Positive effect on	Social presence	Social elements
Chaves et al. (2018)	16_1	Invisible	Personality	Positive effect on	Social presence	Social elements
Chaves et al. (2018)	16_2	Invisible	Chronemics	Negative effect on	Usefulness	Functional elements
Chaves et al. (2018)	16_3	Verbal	Style	Positive effect on	Usefulness	Functional elements
Kim et al. (2018)	18_1	Verbal	Style	Positive effect on	Social presence	Social elements
Kim et al. (2018)	18_3	Verbal	Adaptivity	Positive effect on	Trust	Relational elements
Kim et al. (2018)	18_4	Verbal	Content	Positive effect on	Trust	Relational elements
Huber et al. (2018)	24_1	Visual	CMC	Positive effect on	Social presence	Social elements
Huber et al. (2018)	24_2	Visual	CMC	Positive effect on	Usefulness	Functional elements
Huber et al. (2018)	24_3	Visual	CMC	Positive effect on	Usefulness	Functional elements
Huber et al. (2018)	24_4	Visual	CMC	Positive effect on	Usefulness	Functional elements
Huber et al. (2018)	24_5	Visual	CMC	Positive effect on	Usefulness	Functional elements
Huang et al. (2018)	32_1	Invisible	Intelligence	No effect on	Usefulness	Functional elements
Schuetzler et al. (2018)	43_1	Visual	Agent appearance	No effect on	Rapport	Relational elements
Schuetzler et al. (2018)	43_2	Verbal	Adaptivity	Positive effect on	Rapport	Relational elements
Schuetzler et al. (2018)	43_3	Visual	Agent appearance	Negative effect on	Rapport	Relational elements
Kim et al. (2018)	57_1	Invisible	Haptics	Positive effect on	Usefulness	Functional elements
Miehle et al. (2018)	61_1	Interaction	Mode	Positive effect on	Usefulness	Functional elements
Miehle et al. (2018)	61_2	Interaction	Mode	Positive effect on	Rapport	Relational elements
Miehle et al. (2018)	61_3	Interaction	Mode	Positive effect on	Usefulness	Functional elements
Miehle et al. (2018)	61_4	Interaction	Mode	Positive effect on	Usefulness	Functional elements
Miehle et al. (2018)	61_6	Interaction	Mode	Positive effect on	Rapport	Relational elements
Kang et al. (2018)	86_1	Verbal	Style	Positive effect on	Trust	Relational elements
Kang et al. (2018)	86_10	Verbal	Style	No effect on	Usefulness	Functional elements
Kang et al. (2018)	86_12	Verbal	Style	No effect on	Usefulness	Functional elements
Kang et al. (2018)	86_2	Verbal	Style	Positive effect on	Rapport	Relational elements
Kang et al. (2018)	86_3	Verbal	Style	Positive effect on	Trust	Relational elements
Kang et al. (2018)	86_4	Verbal	Style	Positive effect on	Rapport	Relational elements
Kang et al. (2018)	86_5	Verbal	Style	Positive effect on	Social presence	Social elements
Kang et al. (2018)	86_7	Verbal	Style	Positive effect on	Usefulness	Functional elements
Kang et al. (2018)	86_8	Verbal	Style	No effect on	Trust	Relational elements
Kang et al. (2018)	86_9	Verbal	Style	No effect on	Rapport	Relational elements
Pecune et al. (2018)	92_1	Invisible	Chronemics	Negative effect on	Rapport	Relational elements
Pecune et al. (2018)	92_2	Verbal	Adaptivity	Negative effect on	Rapport	Relational elements
Gnewuch et al. (2018)	93_1	Invisible	Chronemics	Positive effect on	Perceived humanness	Social elements
Gnewuch et al. (2018)	93_2	Invisible	Chronemics	Positive effect on	Social presence	Social elements
Gnewuch et al. (2018)	93_3	Invisible	Chronemics	Positive effect on	Usefulness	Functional elements
Lee et al. (2019)	10_1	Verbal	Adaptivity	Positive effect on	Rapport	Relational elements
Lee et al. (2019)	10_2	Verbal	Style	Negative effect on	Usefulness	Functional elements
Lee et al. (2019)	10_3	Verbal	Adaptivity	Positive effect on	Rapport	Relational elements
Narducci et al. (2019)	101_1	Interaction	Degree of freedom	Positive effect on	Usefulness	Functional elements
Narducci et al. (2019)	101_2	Interaction	Degree of freedom	Positive effect on	Usefulness	Functional elements
Benlian et al. (2019)	108_1	Invisible	Chronemics	Positive effect on	Trust	Relational elements
Benlian et al. (2019)	108_2	Visual	Proxemics	Positive effect on	Trust	Relational elements
Benlian et al. (2019)	108_3	Verbal	Content	Positive effect on	Trust	Relational elements
Pfeuffer et al. (2019)	109_1	Visual	Agent appearance	Positive effect on	Trust	Relational elements
Pfeuffer et al. (2019)	109_2	Visual	Agent appearance	No effect on	Social presence	Social elements
Pfeuffer et al. (2019)	109_3	Visual	Agent appearance	Positive effect on	Rapport	Relational elements
Kim et al. (2019)	11_1	Verbal	Adaptivity	Positive effect on	Usefulness	Functional elements
Kim et al. (2019)	11_2	Verbal	Style	Positive effect on	Usefulness	Functional elements
Yang et al. (2019)	13_1	Verbal	Style	Positive effect on	Usefulness	Functional elements
Yang et al. (2019)	13_2	Interaction	Mode	Negative effect on	Trust	Relational elements
Yang et al. (2019)	13_5	Invisible	Chronemics	Positive effect on	Usefulness	Functional elements
Yang et al. (2019)	13_6	Verbal	Style	Negative effect on	Social presence	Social elements
Jeong et al. (2019)	15_1	Interaction	Degree of freedom	Negative effect on	Social presence	Social elements
Jeong et al. (2019)	15_2	Interaction	Degree of freedom	Negative effect on	Trust	Relational elements
Jeong et al. (2019)	15_3	Interaction	Degree of freedom	Positive effect on	Rapport	Relational elements
Lee et al. (2019)	17_1	Auditory	Voice qualities	Effect on	Social presence	Social elements
Lee et al. (2019)	17_2	Visual	Agent appearance	Positive effect on	Social presence	Social elements
Akahori et al. (2019)	28_1	Verbal	Content	Positive effect on	Usefulness	Functional elements
Akahori et al. (2019)	28_2	Invisible	Chronemics	No effect on	Usefulness	Functional elements
Akahori et al. (2019)	28_3	Interaction	Mode	No effect on	Usefulness	Functional elements
Akahori et al. (2019)	28_4	Interaction	Mode	Positive effect on	Usefulness	Functional elements
Akahori et al. (2019)	28_5	Verbal	Content	No effect on	Usefulness	Functional elements
Akahori et al. (2019)	28_6	Interaction	Mode	No effect on	Usefulness	Functional elements
Akahori et al. (2019)	28_7	Interaction	Mode	No effect on	Usefulness	Functional elements
Cho (2019)	33_1	Interaction	Mode	Positive effect on	Social presence	Social elements
Cho (2019)	33_2	Interaction	Mode	No effect on	Social presence	Social elements
Mu et al. (2019)	38_1	Interaction	Degree of freedom	Negative effect on	Usefulness	Functional elements
Mu et al. (2019)	38_2	Interaction	Degree of freedom	No effect on	Usefulness	Functional elements
Mu et al. (2019)	38_3	Interaction	Degree of freedom	Negative effect on	Usefulness	Functional elements
Mu et al. (2019)	38_4	Interaction	Degree of freedom	Positive effect on	Usefulness	Functional elements
Clark et al. (2019)	4_1	Verbal	Style	Positive effect on	Rapport	Relational elements
Clark et al. (2019)	4_2	Verbal	Content	Positive effect on	Rapport	Relational elements
Clark et al. (2019)	4_3	Verbal	Content	Positive effect on	Usefulness	Functional elements
Clark et al. (2019)	4_4	Verbal	Content	Positive effect on	Usefulness	Functional elements
Clark et al. (2019)	4_5	Verbal	Style	No effect on	Rapport	Relational elements
Winkler et al. (2019)	5_3	Invisible	Chronemics	Positive effect on	Rapport	Relational elements
Ashktorab et al. (2019)	6_1	Visual	CMC	Positive effect on	Usefulness	Functional elements
Ashktorab et al. (2019)	6_2	Verbal	Content	Positive effect on	Usefulness	Functional elements
Ashktorab et al. (2019)	6_3	Verbal	Content	Negative effect on	Usefulness	Functional elements
Ashktorab et al. (2019)	6_4	Verbal	Style	Positive effect on	Usefulness	Functional elements
Yu et al. (2019)	64_1	Interaction	Mode	Positive effect on	Rapport	Relational elements
Yu et al. (2019)	64_2	Auditory	Voice qualities	Negative effect on	Rapport	Relational elements
Chin et al. (2019)	8_1	Verbal	Style	Negative effect on	Rapport	Relational elements
Chin et al. (2019)	8_2	Verbal	Style	Positive effect on	Rapport	Relational elements
Chin et al. (2019)	8_3	Verbal	Style	Positive effect on	Usefulness	Functional elements
Chin et al. (2019)	8_4	Verbal	Adaptivity	Negative effect on	Usefulness	Functional elements
Chin et al. (2019)	8_5	Verbal	Style	Negative effect on	Rapport	Relational elements
Chin et al. (2019)	8_6	Verbal	Adaptivity	Positive effect on	Usefulness	Functional elements
Schuetzler et al. (2019)	80_1	Invisible	Intelligence	Negative effect on	Usefulness	Functional elements
Schuetzler et al. (2019)	80_2	Invisible	Intelligence	Negative effect on	Rapport	Relational elements
Schuetzler et al. (2019)	80_3	Invisible	Intelligence	No effect on	Rapport	Relational elements
Skjuve et al.(2019)	81_2	Visual	Appearance	No effect on	Trust	Relational elements
Skjuve et al.(2019)	81_3	Visual	Appearance	Positive effect on	Perceived humanness	Social elements
Westerman et al. (2019)	82_1	Visual	CMC	Negative effect on	Perceived humanness	Social elements
Westerman et al. (2019)	82_2	Visual	CMC	Negative effect on	Usefulness	Functional elements
Westerman et al. (2019)	82_3	Visual	CMC	No effect on	Perceived humanness	Social elements
Westerman et al. (2019)	82_4	Visual	CMC	No effect on	Usefulness	Functional elements
Westerman et al. (2019)	82_5	Visual	CMC	Negative effect on	Social presence	Social elements
Westerman et al. (2019)	82_6	Visual	CMC	No effect on	Social presence	Social elements
Westerman et al. (2019)	82_7	Visual	CMC	Negative effect on	Rapport	Relational elements
Westerman et al. (2019)	82_8	Visual	CMC	No effect on	Rapport	Relational elements
Diederich et al. (2019)	83_1	Interaction	Degree of freedom	No effect on	Usefulness	Functional elements
Diederich et al. (2019)	83_2	Interaction	Degree of freedom	Negative effect on	Social presence	Social elements
Diederich et al. (2019)	83_3	Interaction	Degree of freedom	Negative effect on	Perceived humanness	Social elements
Hoegen et al. (2019)	84_1	Verbal	Adaptivity	No effect on	Trust	Relational elements
Hoegen et al. (2019)	84_2	Verbal	Adaptivity	Positive effect on	Trust	Relational elements
Hoegen et al. (2019)	84_3	Verbal	Adaptivity	No effect on	Trust	Relational elements
Hoegen et al. (2019)	84_4	Verbal	Adaptivity	No effect on	Usefulness	Functional elements
Nordheim et al. (2019)	85_1	Invisible	Personality	Positive effect on	Trust	Relational elements
Nordheim et al. (2019)	85_2	Invisible	Personality	No effect on	Trust	Relational elements
Nordheim et al. (2019)	85_3	Invisible	Personality	No effect on	Trust	Relational elements
Kontogiorgos et al.(2019)	90_1	Visual	Agent appearance	Positive effect on	Rapport	Relational elements
Kontogiorgos et al.(2019)	90_2	Visual	Agent appearance	Negative effect on	Usefulness	Functional elements
Kontogiorgos et al.(2019)	90_3	Interaction	Mode	No effect on	Rapport	Relational elements
Iovine et al. (2020)	100_1	Interaction	Degree of freedom	Positive effect on	Usefulness	Functional elements
Iovine et al. (2020)	100_2	Interaction	Degree of freedom	Positive effect on	Usefulness	Functional elements
